# Increase in Breastfeeding Rates in Baby-Friendly Hospitals in Greece: Comparison with the National Study of 2017

**DOI:** 10.3390/children11080932

**Published:** 2024-07-31

**Authors:** Evangelos Christou, Eftychia Liakou, Abraham Pouliakis, Rozeta Sokou, Paraskevi Volaki, Styliani Paliatsou, Theodora Boutsikou, Nicoletta Iacovidou, Zoi Iliodromiti

**Affiliations:** 1 Neonatal Department, Aretaieio Hospital, National and Kapodistrian University of Athens, 115 28 Athens, Greece; eliakou73@yahoo.gr (E.L.); sokourozeta@yahoo.gr (R.S.); v.volaki@hotmail.com (P.V.); theobtsk@gmail.com (T.B.); niciac58@gmail.com (N.I.); ziliodromiti@yahoo.gr (Z.I.); 2 2nd Department of Pathology, “ATTIKON” University Hospital, National and Kapodistrian University of Athens, 124 61 Athens, Greece; apou1967@gmail.com

**Keywords:** exclusive breastfeeding, neonate, baby-friendly hospital

## Abstract

Background/Objectives: The primary purpose of this study was to correlate the factors influencing the onset and duration of exclusive breastfeeding (EBF) and breastfeeding (BF) rates at different time points in baby-friendly hospitals (BFHs) in Greece. Methods: This study was conducted from October 2020 to January 2022. The sample consisted of 1201 mothers with corresponding newborn births out of the total 7201 that took place during the same period. We used a questionnaire that the mothers answered during the first hour after birth, before being discharged from the maternity hospital, and at the 2nd, 4th, and 6th month of the infant’s life. The results were compared with the data of the national study from 2017 (concerning births from general maternity hospitals and not only from BFHs). Results: The EBF rate within 1 h after birth was 71.3%, which gradually declined to 21.2% in the 6th month. Between the two studies, differences were recorded in BF and EBF rates at the 6th and 4th month of the infants’ life, respectively, especially in mothers who are less likely to breastfeed (e.g., those without breastfeeding experience, those over 40 years old, etc.). Conclusion: This study showed that EBF and BF rates are higher in infants born in BFHs compared to non-BFHs both during their hospitalization in the first days of life and during the first 6 months of life. BFHs also play a decisive role in mothers who, due to their socio-economic profile, have lower chances of starting to breastfeed.

## 1. Introduction

Breastfeeding remains one of the most important intervention factors to ensure public health worldwide. A low breastfeeding rate is a problem of upper–middle-income countries, and according to the 2019 available data, the rate of exclusive breastfeeding (EBF) was 37% [1], standing in contrast to that of low- and middle-income countries, which was 44%, as reported by the UNICEF study for the period 2014–2020 [2].

In Greece, the latest available data on breastfeeding rates are from the 2017 National Study by the Institute of Child Health (ICH) [3], which reported an increase in breastfeeding rates during the first 6 months of life, compared to that of the 2007 respective National Study (45% vs. 22%) [4]. An improvement in breastfeeding practices, including an increase in the initiation of breastfeeding within the 1st hour after birth, rooming-in 24 h per day and reductions in formula milk administration were also reported. However, at the end of the 6th month, a reduced incidence of EBF rates was observed (<1%). 

The COVID-19 pandemic affected the breastfeeding indicators in Greece. Tigka et al. reported that the EBF rate reached 7.2% during the 6th month, which is higher than that reported in studies conducted in the pre-COVID-19 era [5]. Despite the improvement observed in breastfeeding indicators, as established by the WHO [6], the pandemic imprint on the total breastfeeding process remains doubtful. 

The WHO and UNICEF established “The Baby Friendly Hospitals (BFH) initiative” in 1991 in an attempt to protect, promote and support breastfeeding [7]. In Greece, four public hospitals have been so far characterized as such, including the “Attikon” General Hospital and “Elena Venizelou” Maternity Hospital in 2011, the “Aretaieion” Hospital in 2016 and the General Hospital of “Preveza” in 2019, the first regional hospital that received this accreditation. In Greece, births take place in both public and private maternity hospitals. Previously, there was also one private maternity hospital operating as a BFH, but it had not been re-certified. At the time of study, births in the BFHs constituted approximately 6.5% of the total number of births.

The requirements for a maternity hospital to be characterized as a BFH are as follows: (a) at least 75% of babies delivered in the preceding year must have been exclusively breastfed, or fed exclusively expressed breast milk from birth to discharge, (b) the hospital must be compliant with the International Code of Marketing Breast-milk Substitutes, and (c) the “Ten Steps to Successful Breastfeeding” must be implemented [8]. There is now strong evidence that if an institution is a BFH, it is beneficial to promote breastfeeding. A systematic review showed that skin-to-skin contact of the neonate with the mother increased the rate and duration of breastfeeding [9] and that the implementation of 9 out of the 10 steps in a BFH significantly increased EBF rates at 6 months compared to the control group [10]. 

The primary aim of this study was to correlate factors influencing the onset and duration of EBF and breastfeeding at different time points, and their effect on the BFH environment. The secondary aim was to compare these determinants with the study, thus offering the latest epidemiological data on breastfeeding in Greece for the first time in maternity hospitals [3]. 

## 2. Methods

### 2.1. Study Design

This prospective observational study was conducted from October 2020 to January 2022 during the public health emergency measures due to the COVID-19 pandemic. It was approved by the scientific boards of the 4 participating hospitals, the Aretaieio Hospital, National and Kapodistrian University of Athens (219/18-06-20), the “Attikon” General Hospital, National and Kapodistrian University of Athens (479/24-08-20), the “Elena Venizelou” Maternity Hospital, Athens (19608/03-09-20), and the General Hospital of “Preveza” (62/29-09-20). The first 3 are located in Athens, and the last one is located in the west part of Greece. These hospitals are the only public institutions that were characterized as BFHs by the Ministry of Health. The study was conducted based on the STROBE guidelines and the Declaration of Helsinki (Supplementary Material) [11]. 

### 2.2. Sample

The recruited mothers gave birth in the above-mentioned hospitals and were hospitalized in the postnatal wards. Requirement for inclusion in the study was the mother’s availability for telephone interviews at the predetermined time points. The study sample consisted of 1201 mothers who delivered 1201 newborns out of a total of 7101 births that were registered in these hospitals during the corresponding time period. The initial sample size was based on power analysis of the population of the individual hospitals from the previous year and was calculated to obtain a margin of error of ≤5%. In the study, due to changes in the number of women managed by each hospital, the final population and sample size were different. However, the margin of error for each hospital in the study was 4%, 4%, 5% and 3%, respectively, for the participating hospitals, which is considered adequate, and thus the sample of 1201 women is representative of the population of 7201 women with a margin of error of ≤5%. 

The questionnaire was checked for validity and reliability in order to make appropriate corrections to its content through a pilot interview with 20 mothers who were not included in the final sample. Mothers younger than 15 years of age, with significant health problems during pregnancy or postpartum that affected their ability to breastfeed, those whose newborns experienced major health problems or significant congenital anomalies, and finally those with multiplet pregnancies were excluded from the study. 

### 2.3. Data Collection

Participation was on a volunteer basis and mothers were informed and signed a consent prior to recruitment. The structured interview process involved the following 5 time points: the 1st hour following birth, prior to discharge, at the end of the 2nd, 4th and 6th month of life. At the first two time points, interviews were conducted through a face-to-face interview by a researcher. At the subsequent three time points, the interviews were conducted over the phone. The average duration was 10 min. 

The questionnaire included open- and close-ended questions, and participants could answer either between dichotomized answers (YES/NO), through a list of different possible answers, or through open-field answers. 

Stratified random sampling was used to improve the precision of the study. The face-to-face interview and the telephone interview were conducted by 2 researchers, with previous experience in this kind of interview process.

### 2.4. Variables 

The questionnaire was original and created by the main investigator. The first part included 24 questions on the obstetric history of the index pregnancy, the second part included 8 questions on the perinatal history and the last part included 11 questions on the mother’s medical history. The choice of the variables examined was based on previous similar studies [12,13] and on specific factors existing in Greece that influence breastfeeding behaviours. The COVID-19 pandemic modified the design of the questionnaire. Finally, we attempted to investigate all confounding factors that could affect the main results of the study. 

### 2.5. Indicators of Breastfeeding

The definitions of breastfeeding that we used in our study were those given by the World Health Organization [6] and are shown below:

1. Exclusive breastfeeding (EBF) refers to breastfeeding only with breast milk and without any other form of foods or liquids, with the exception of oral rehydration solutions, drops, and syrups (vitamins, minerals, medicines).
2. Breastfeeding (BF) refers to ‘any breastfeeding’, i.e., the mode of feeding infants who receive breast milk with or without any other type of food or drink, including breast milk substitutes (non-human milk and formula).

### 2.6. Statistical Analysis

Data were collected using a Microsoft Excel version 2023 file (Microsoft Corporation, Redmond, WA, USA), with each questionnaire in a row and each variable in a column to facilitate subsequent data processing. Statistical analysis was performed within the environment of the SAS software platform (version 9.4, SAS Institute Inc., Cary, NC, USA). Since all variables were categorical, the descriptive characteristics were presented as frequencies and the relevant percentages. The chi-square test was applied for comparisons among groups, and for comparisons of percentages, the test of proportions was applied. All tests were two-sided and the study significance level was set as *p* < 0.05.

## 3. Results

**Figure 1:**
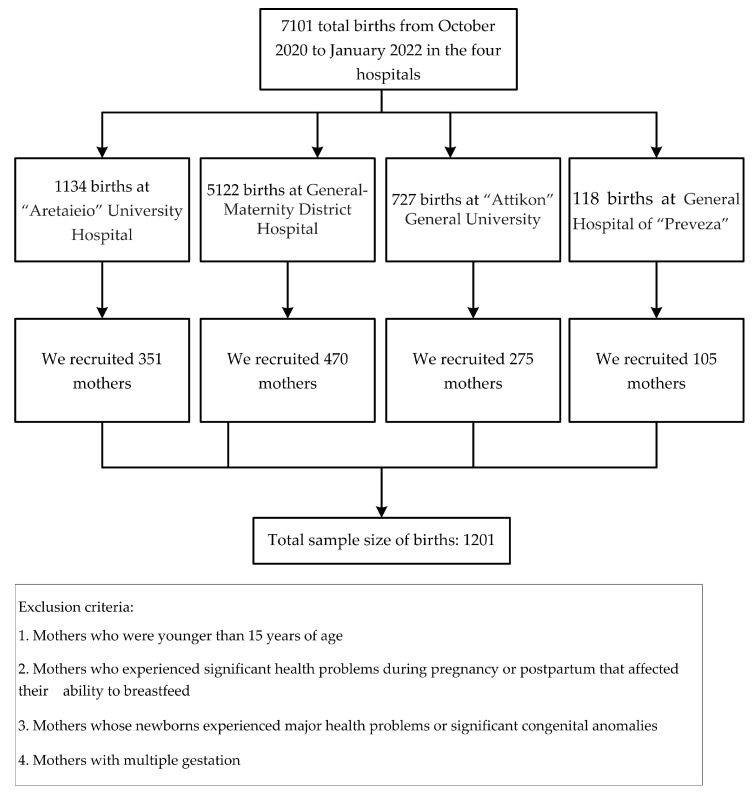
Participant flow.

Figure 1 presents the flow chart for the study sample size. In total, 1201 mothers participated in the study.

### 3.1. Infant Feeding Rates

**Figure 2:**
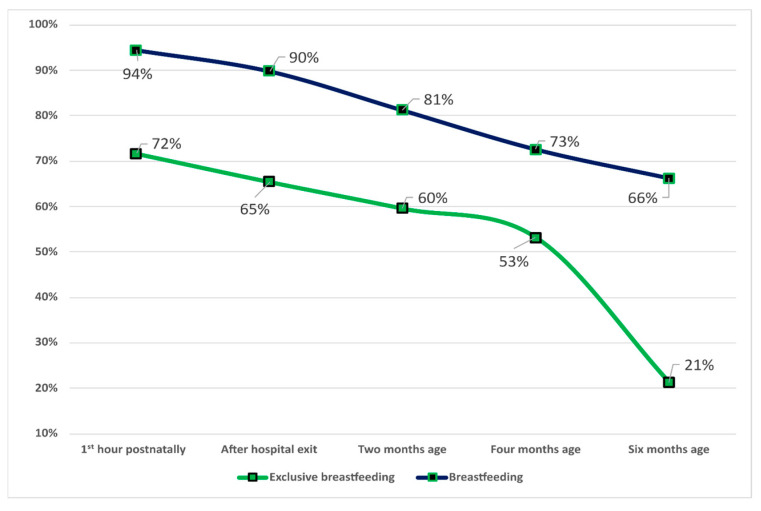
Rates of EBF and BF at different study points at BFHs in Greece.

Table 1 presents the main characteristics of the sample, including mothers and newborns. Regarding the type of infant feeding, there is a gradual decline in EBF within the 1st hour after birth up to the 6th month of life. The EBF rate was 71.7% in the 1st hour after birth, 65.5% immediately before leaving the maternity hospital, 59.6% in the 2nd month, 53.2% in the 4th month and 21.2% in the 6th month. BF rates decrease less over time compared to EBF rates. BF rates at the 1st hour postpartum were 94.5%, and 66.2% at the 6th month. Figure 2 shows details on the percentage of infants fed EBF and BF. 

In total, 339 mothers did not breastfeed their infants during their hospitalization in the maternity hospital. Overall, 81 (23.9%) stated that the reason was their own inability to breastfeed adequately, 64 (18.9%) stated that their baby could not breastfeed, 19 (5%) claimed it was due to medical reasons that did not allow them to breastfeed their baby, 30 (8.8%) claimed it was due to medical reasons associated with the newborn and 90 (26.5%) gave multiple reasons, while undetermined reasons were described by the remaining 55 mothers (16.2%). 

### 3.2. Factors Associated with EBF and BF Rates

Various factors related to individual maternal, perinatal and obstetric history of pregnancy were analyzed to determine their influence on the feeding pattern of infants born in BFHs.

Regarding the rates of EBF and the level of maternal education at all time points, those with post-secondary education compared to those with basic and/or secondary education had higher EBF rates (*p* < 0.005). At all time points, those who had previous breastfeeding experience had increased EBF rates compared to those who breastfed for the 1st time. The results were similar for those mothers who belonged to a structured family and were not single parents, as they had statistically increased EBF rates compared to mothers who were single parents and did not have support from a partner. Mothers of non-Greek origin reported increased EBF rates compared to Greek mothers. Mothers with a family income > EUR 12,000 had increased EBF rates compared to those with a family income < EUR 12,000. No difference was recorded between those who had maternal leave during the 6-month breastfeeding period and those who did not at any time point (*p* > 0.005). Finally, full-term infants had increased EBF rates compared to preterm infants (<37 weeks of gestation) as well as those born with a weight >2500 gr compared to those who had low birth weight (<2500 gr). Table 2 presents the results in detail.

The results regarding BF rates and the effect of the above factors analyzed for their relationship with EBF were slightly different. More specifically, mothers with higher education compared to graduates of basic/secondary education showed increased BF rates at all time points (*p* < 0.001), which decreased with the increase in the infant’s age. Working mothers, with the exception of the 1st hour after birth (*p* = 0.4) and immediately after discharge (*p* = 0.02), achieved increased BF rates compared to mothers who were not working. This also applies to the decrease rate over time. In contrast to the positive effect of previous breastfeeding experience on EBF rates at all five consecutive time points, it played a positive role in BF only in the 4th (*p* = 0.002) and 6th month (*p* = 0.003). Raising the infant with the support of a partner and not as a single parent also increased the BF rates at all time points. At all time points, non-Greek mothers recorded higher BF rates vs Greek mothers, as in the case of the EBF rates. A family income > EUR 12,000 also played a decisive role in the promotion of BF as well as EBF compared to those with a lower income. Finally, neonatal factors such as gestational age and birth weight seemed to play a significantly minor role in BF rates compared to EBF rates. Table 3 presents the results in detail. 

### 3.3. Comparison with the Results of the Institute of Child Health National Breastfeeding Study

The National Breastfeeding Study [3], published in 2017, was conducted by the ICH under the auspices of the National School of Public Health. In total, 43 maternity hospitals from all geographical regions of the country were included in the study involving 870 infants, and the main objectives included the recording of breastfeeding rates, calculation of breastfeeding indicators and investigation of determinants of breastfeeding frequency. The comparison included the determinants for EBF at the 4th month and BF at the 6th month, as only at these time points was it possible to compare. 

**Figure 3:**
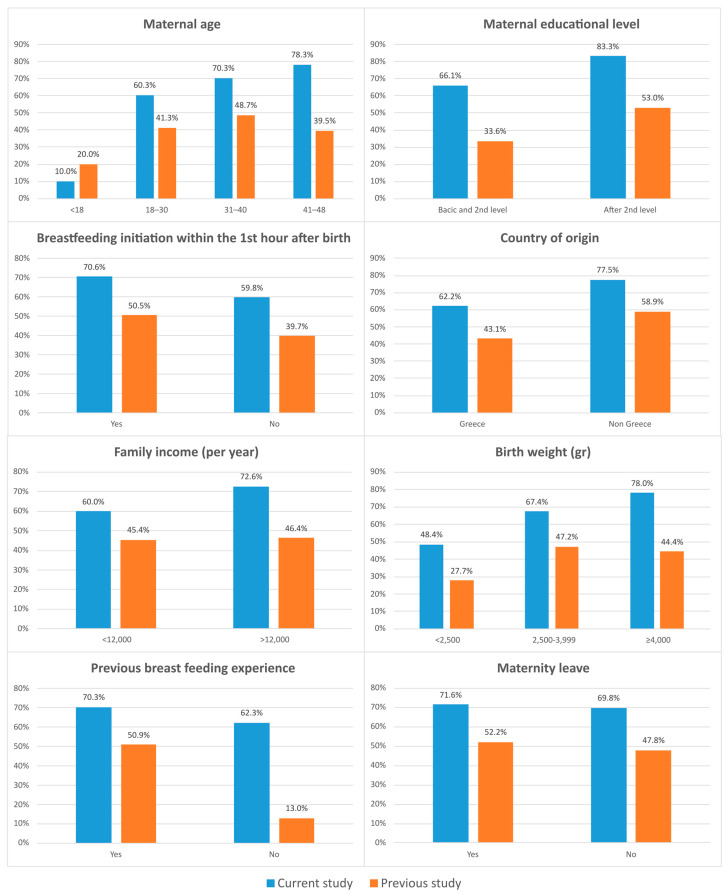
Comparison of BF rates at six months between our study and the 2017 national study based on various factors of influence on breastfeeding.

**Figure 4:**
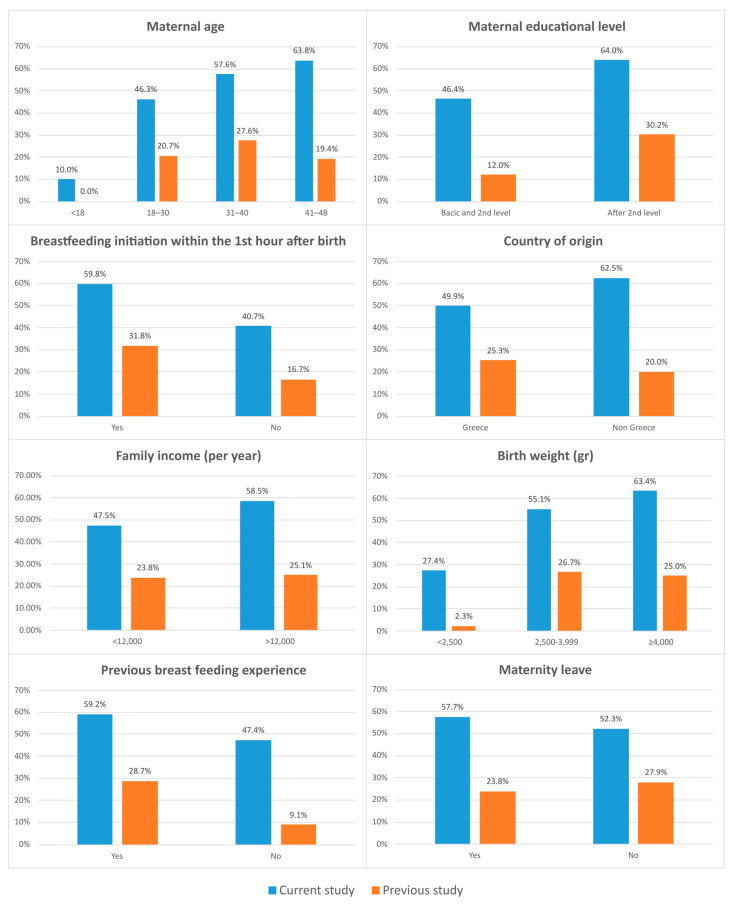
Comparison of EBF rates at four months between our study and the 2017 national study based on various factors of influence on breastfeeding.

The important differences recorded in BF rates at the 6th month of the infants’ life were among women who had no previous breastfeeding experience (62.3% vs. 13%), those with basic and intermediate education (66.1% vs. 33.6%) and those in the 41–48 age group (78.3% vs. 39.5%). Detailed results are presented in Figure 3. In relation to EBF rates at the 4th month, the differences recorded were also significant and were mainly detected in those who had no previous breastfeeding experience (47.4 vs. 9.1%), in newborns with a BW >4000 g (63.4% vs. 25%), in non-Greek mothers (62.5% vs. 20%) and in those in the 41–48 age group (63.8 vs. 19.4%). Detailed results are presented in Figure 4.

## 4. Discussion

The BFHs create a favorable environment for breastfeeding mothers and their infants, leading to better breastfeeding rates compared to non-BFHs, and promote optimal breastfeeding practices. In the national study of 2017, the rates of EBF during the first 24 h of life were 66% and declined to 51% at the end of the first week. This decline continued, reaching 40% at the end of the 1st month of life, 25% at the end of the 4th month of life, and finally 0.8% at the end of the 6th month of life. As previous studies [14,15] reported that the education of healthcare professionals and parents has a positive impact on EBF and BF rates, we collected data from infants born in BFHs and studied the determinants, set by the WHO, as well as the practices influencing the promotion of EBF and BF.

The main research goals of our study were (a) to record for the first time EBF and BF rates in BFHs, (b) the assessment of the applied breastfeeding practices in BFHs and their effect on the EBF and BF rates, and (c) finally to compare the results of our study with the National Breastfeeding Study of 2017. The main determinants studied were maternal education level, maternal work during the breastfeeding period, previous breastfeeding experience, single-parent household, maternal ethnic origin, family income status, birth weight and gestational age of the newborn in the BFHs. 

The results of the current study demonstrated clearly better frequency of EBF and BF compared to the national study of 2017. The EBF rate within the 1st hour after the birth was 94%, while in the national study of 2017, it was 65.7%. This difference is attributed to skin-to-skin contact recorded more often in BFHs compared to non-BFH. Data from a national study of Brazil showed a significant predominance in EBF initiation rates in mothers who gave birth in public, private, or mixed BFHs vs. non-BFHs (85.3% versus 72.4%) [16]. Similarly, the findings from four public hospitals in Hong Kong showed that 64.6% of the newborns initiated breastfeeding within the 1st hour after labor when the hospitals participated in the Baby-Friendly Initiative, compared to 40.9% of the newborns before the BFI [17].

The difference in EBF rates at subsequent time points was also significant, as at the 2nd month, it was 59.6% vs. 34.1% as reported in the 2017 national study; at the 4th month, it was 53.2% vs. 24.5%; at the 6th month, it was 21.2% vs. 0.8%. In line with the differences in EBF rates between the two studies, the rates of BF were increased at all time points in the present study, except for after birth, where the rates in both studies fluctuated around 94%. At the 6th month, 66.2% of infants were breastfeeding compared to 45.3% in the 2017 national study.

The importance of the higher educational profile of breastfeeding mothers was confirmed by our study. Studies from Europe [18] and from low-income countries [19] showed that women of lower education are more likely to stop breastfeeding early, to breastfeed for a shorter period of time, or to exhibit behaviors that may be not beneficial to breastfeeding. The impact of previous breastfeeding experience on the rates of mainly EBF rather than BF was reflected in our results. The more experienced the mother with previous breastfeeding was, the greater ease of coping with possible difficulties that may arise during the process, and the better the management of emotional distress was. It is important to emphasize that mothers who had breastfed for a short period of time in the past do not seem to be in an advantageous position compared to those who had not breastfed [20]. EBF and BF rates were also significantly increased in breastfeeding women with supportive partners. It is known that the partner’s influence plays an important role in the initiation, exclusivity and duration of breastfeeding [21], due to the ability to provide psychological support to the mother, to make the mother more relaxed and committed to breastfeeding, and actively participate in the process [22]. An interesting finding is that non-Greek mothers had increased EBF and BF rates compared to Greek women, which is probably due to the breastfeeding culture that prevails in the country of origin, despite their financial status. With the exception of a few time points, a higher family income seemed to help both EBF and BF. As already reported in the literature, breastfeeding women from developed and developing countries with a lower economic status who are obliged to work longer hours have less time to devote to breastfeeding, greater family difficulties, less support from their partners and increased stress levels, resulting in less breastfeeding and shorter breastfeeding durations [23].

A significant difference emerges between the reported causes for the cessation of breastfeeding. Despite the fact that in our study and in that of 2017 as well, the main cause were problems related to the mother, such as insufficient milk production, nipple injury and fatigue or depression; in our study, the reasons concerning the newborn were significantly increased compared to that reported in 2017 (18.9% vs. 9.2%). Training of health professionals dealing with breastfeeding to prepare parents for breastfeeding, avoidance of unnecessary administration of nutritional supplements to breastfeeding infants without medical reason, pre-natal and post-natal preparation classes for mothers, their support from specialists to immediately deal with problems that may arise during breastfeeding, and above all the increased mother–infant contact immediately after birth with skin-to-skin that continues during the whole duration of their stay in the hospital, in addition to the 24 h rooming-in of mothers and infants in the postnatal ward, offered special benefits in the promotion of breastfeeding [24,25,26].

An important finding is that the rates of EBF at the 4th month and BF at the 6th month were significantly increased in our study in relation to the national study of 2017, especially in mothers who based on their demographic characteristics are less likely to breastfeed. The effect of BFHs was decisive in breastfeeding women aged 41–48 who faced significant problems related to lactogenesis or other health problems that may inhibit the initiation and duration of breastfeeding [27]. The results at the same time points were similar for mothers who had no previous breastfeeding experience, for those who gave birth to newborns with increased birth weight, or were likely to have health problems after birth, resulting in separation from their mothers during the critical time for the beginning of breastfeeding, and for those with a lower level of education.

The emergency measures taken at the beginning of the COVID-19 pandemic negatively affected the breastfeeding process. In the first period of the COVID-19 pandemic, the rates of breastfeeding declined due to unclear recommendations on breastfeeding from scientific societies [28,29,30,31,32], public health measures, and the separation of neonates from their mothers in cases of maternal COVID-19 infection [33]. During this period, we observed a similar decline in EBF rates at discharge in comparison with the expected rates in BFH (65.5%), a finding that is in line with other studies [34,35]. After this period, due to different interventions, we observed an increase mainly in the duration but not in the rate of breastfeeding [36]. 

Our study had some limitations. First, the sample of the study involves three hospitals in the capital of the country, and only one from the periphery, as only these are characterised as BFHs. A second limitation is that despite the fact that the WHO-based breastfeeding indicators were used, there were several difficulties in comparison with other studies that did not use the same ones. The third limitation is the sample selection, since the breastfeeding process involves increased stress for mothers and reluctancy to participate. However, only one mother during the study withdrew from the study as her infant passed away. The language communication problems encountered with the non-Greek breastfeeding mothers were resolved with the full support of the hospitals’ translation service. Additionally, the time points and factors where the correlation between the two studies was observed emerged randomly (as they were studied in both), making a more detailed comparison not feasible. However, this was unavoidable due to the way they were designed. Finally, the COVID-19 pandemic caused several difficulties regarding the recruitment of participants, and, as a result, the total sample could not be larger.

## 5. Conclusions

This study showed that EBF and BF rates are higher in infants born in BFHs compared to non-BFHs both during their hospitalization in the first days of life and during the first 6 months of life. BFHs also play a decisive role in mothers who, due to their socio-economic profile, have lower chances of starting to breastfeed and to breastfeed for an extended period. 

In the future, health authorities should support the establishment of new BFHs, further support existing ones, and create a protective framework to support breastfeeding in order for Greece to approach the WHO’s declared targets on EBF for 2025. Furthermore, financial and administrative support from the government to maternity wards with higher EBF and BF rates is essential, with long-term benefits for public health and the financial well-being of the state.

## Figures and Tables

**Table 1:** Baseline characteristics of the sample population.

Variable	N (%)
Age of mother (years)	
15–17	20 (1.6%)
18–30	422 (35.3%)
31–40	685 (57.3%)
41–48	70 (5.8%)
Gravida	
Primigravida	560(46.6%)
No primigravida	640 (53.4%)
Mode of delivery	
Vaginal delivery	732 (61%)
Cesarean section	468 (39%)
Marital status	
Non-married	119 (9.9%)
Married	1081 (90.1%)
Previous breastfeeding experience	
Yes	608 (50.6%)
No	592 (49.4)
Educational status	
Primary School	106 (8.8%)
High school	568 (47.4%)
University	524 (43.8%)
Employment during breastfeeding period	
Yes	645 (53.7%)
No	555 (46.3%)
Baby weight (g)	3140

**Table 2:** EBF rates in relation to maternal and infant socioeconomic factors at the study time points.

	Study Time Point									
	1st Hour Following Birth		After Discharge		Two Months of Life		Four Months of Life		Six Months of Life	
	%	*p*	%	*p*	%	*p*	%	*p*	%	*p*
Maternal educational level										
Primary/High School	67.4%	<0.01	60.1%	<0.01	52.3%	<0.01	46.4%	<0.01	18.6%	0.04
University	79.2%		74.6%		71.1%		64%		25.5%	
Maternal employment during breastfeeding period										
Yes	73.6%	0.11	69%	0.01	63.6%	0.02	57%	0.04	21.3%	0.96
No	69.5%		61.4%		55%		48.8%		21.2%	
Previous experience of breastfeeding										
Yes	78.7%	<0.01	72.1%	<0.01	65%	0.02	59.2%	<0.01	26.3%	<0.01
No	65%		59%		54.4%		47.4%		16.3%	
Single parent household										
No	73.7%	<0.01	68.1%	<0.01	62.5%	<0.01	56.1%	<0.01	22.1%	0.03
Yes	53.8%		42%		33.1%		27.1%		13.6%	
Country of origin										
Greek	69.7%	<0.01	63.1%	0.02	56.5%	0.02	49.9%	<0.01	18.4%	<0.01
Other	77.5%		72.1%		68.3%		62.5%		29.2%	
Family income										
≤EUR 12,000	67.7%	<0.01	59.1%	<0.01	52.1%	<0.01	47.5%	0.02	19.2%	0.07
>EUR 12,000	76%		71.8%		66.8%		58.5%		23.5%	
Maternity leave										
Yes	73.8%	0.86	70.2%	0.15	64.7%	0.2	58%	0.26	22%	0.37
No	73%		63%		58%		52%		18%	
Birth weight										
<2500 g	40%	<0.01	35.8%	<0.01	32.6%	<0.01	27.4%	<0.01	12.6%	0.03
≥2500 g	74.5%		68%		61.9%		55.4%		21.9%	
Gestational age (GA)										
<37 weeks GA	35.2%	<0.01	34.2%	<0.01	32.4%	<0.01	25.9%	<0.01	7.4%	0.02
≥37weeks GA	75.3%		68.6%		62.3%		55.9%		22.6%	

**Table 3:** BF rates in relation to maternal and infant socioeconomic factors at the study time points.

	Study Time Point									
	1st Hour Following Birth		After Discharge		Two Months of Life		Four Months of Life		Six Months of Life	
	%	*p*	%	*p*	%	*p*	%	*p*	%	*p*
Maternal educational level										
Primary/HighSchool	92.4%	<0.01	87%	<0.01	76.5%	<0.01	66.1%	<0.01	58.4%	<0.01
University	97.9%		95.4%		89.9%		83.3%		78.7%	
Maternal employment during breastfeeding period										
Yes	95%	0.40	91.6%	0.02	84.5%	0.01	77.3%	0.01	71.3%	0.01
No	93.9%		87.7%		77.4%		67.1%		60.4%	
Previous experience of breastfeeding										
Yes	96.4%	0.04	90.9%	0.22	83.9%	0.01	76.6%	0.02	70.3%	0.03
No	92.6%		88.8%		78.6%		68.7%		62.3%	
Single parent household										
No	95%	0.02	91.4%	<0.01	83.8%	<0.01	75.7%	<0.01	69.2%	<0.01
Yes	89.9%		75.6%		57.6%		44.1%		39%	
Country origin										
Greek	93.3%	0.02	87.3%	<0.01	77.6%	<0.01	68.1%	<0.01	62.2%	<0.01
Other	97.8%		96.8%		91.4%		85.1%		77.5%	
Family income										
≤EUR 12,000	93.6%	0.2	86.5%	0.03	75.4%	<0.01	65.8%	<0.01	60%	<0.01
>EUR 12,000	95.3%		92.6%		86.7%		79.5%		72.6%	
Maternity leave										
Yes	94.6%	0.31	91.5%	0.86	84.8%	0.64	78.1%	0.36	71.7%	0.72
No	97%		92%		83%		74%		70%	
Birth weight (gr)										
<2500 g	90.5%	0.07	89.5%	0.91	76.8%	0.25	58.9%	0.01	48.4%	0.01
≥2500 g	94.8%		89.86%		81.6%		73.7%		67.8%	
Gestational age (GA)										
<37 week	91.7%	0.17	90.7%	0.73	72.2%	0.01	57.4%	<0.01	49.1%	<0.01
≥37 week	94.8%		89.7%		82.1%		74.1%		68%	

## Data Availability

Data are contained within the article.

## Supplementary Materials

The following supporting information can be downloaded at: https://www.mdpi.com/article/10.3390/children11080932/s1, STROBE checklist.

## Abbreviations

EBF	Exclusive breastfeeding
BF	Breastfeeding
BFH	Baby-friendly hospital
ICH	Institute of Child Health
